# Nomogram prediction of chronic postsurgical pain in patients with lung adenocarcinoma after video-assisted thoracoscopic surgery: A prospective study

**DOI:** 10.3389/fsurg.2022.1004205

**Published:** 2022-11-09

**Authors:** Wei-can Chen, Li-hong Zhang, Yu-yan Bai, Yi-bin Liu, Jin-wei Liang, He-fan He

**Affiliations:** Department of Anesthesiology, The Second Affiliated Hospital, Fujian Medical University, Quanzhou, China

**Keywords:** nomogram, chronic pain, lung adenocarcinoma, postsurgical pain, video-assisted thoracoscopy

## Abstract

Chronic postsurgery pain (CPSP) refers to persistent or repeated pain around the incision after surgery. Different from acute postoperative pain, the persistence of CPSP seriously affects the quality of life of patients. CPSP has a considerable global impact due to large surgical volumes. Although the development of video-assisted thoracoscopy (VATS) has reduced the risk of CPSP, it still seriously affects patients’ quality of life. Clinical recognition of CPSP at an early stage is limited; therefore, we aimed to develop and validate a nomogram to identify the significant predictive factors associated with CPSP after VATS in patients with lung adenocarcinoma. We screened 137 patients with invasive adenocarcinoma of the lung from among 312 patients undergoing VATS. In this prospective study, patients were divided into the CPSP (*n* = 52) and non-CPSP (*n* = 85) groups according to the occurrence of CPSP. Relevant information was collected 1 day before surgery and 1–3 days after surgery, and the occurrence of CPSP was followed up by telephone at 3 months after surgery. Data on clinical characteristics and peripheral blood leukocyte miRNAs were used to establish a nomogram for predicting CPSP using least absolute shrinkage and selection operator (LASSO) regression methods. The area under curve (AUC) was used to determine the recognition ability of the nomograms. The model was subjected to correction and decision curve analyses. Four variables—body mass index (BMI), history of chronic pain, miR 550a-3p, and visual analog scale (VAS) score on postoperative day 2 (VAS2d)—were selected according to LASSO regression to build the nomogram. The nomogram demonstrated adequate calibration and discrimination in the prediction model, with an AUC of 0.767 (95% confidence interval: 0.679–0.856). The calibration plot showed the best fit between model predictions and practical observations, suggesting that the use of the proposed nomogram to predict CPSP is beneficial. A nomogram consisting of BMI, history of chronic pain, miR 550a-3p, and VAS2d predicted the risk of CPSP after VATS in patients with lung adenocarcinoma.

## Introduction

Chronic postsurgical pain (CPSP) refers to persistent or recurrent pain around the surgical scar for >3 months after surgery (1). In contrast to acute postoperative pain, CPSP presents with uncontrollable persistence, severely affects the patient's quality of life (2, 3), and is extreme enough to provoke severe functional impairment (4). CPSP affects 11.5%–47% of surgical patients (5). Given the considerable number of surgeries performed worldwide, CPSP in patients should not be neglected (6).

Lung cancer is one of the most common malignant diseases, with a high incidence and serious impact on human health and life (7). Surgical resection is the most basic and vital therapy, especially for patients with early-stage lung adenocarcinoma (8, 9). Lobectomy, including open lobectomy and video-assisted thoracoscopic surgery (VATS), is a high-risk procedure that frequently results in CPSP (10–12). As the level of medical care for resectable lung cancer has improved, conventional thoracotomy has been gradually replaced by VATS. In contrast to traditional open lobectomy, VATS lobectomy has the potential for both brief hospital stays and reduced incidence of postoperative complications while guaranteeing therapeutic efficacy (12). Nonetheless, the highest incidence of CPSP can still reach 37.4% with a VATS lobectomy (12). The occurrence of CPSP causes a severe disturbance in patients’ emotional state and sleep quality, leading to a number of psychological issues that immensely impact their prognosis and quality of life (9, 13, 14). Hence, identifying the predictive factors associated with CPSP after VATS will help clinicians achieve targeted prevention and treatment to support patients in re-establishing confidence and improving their quality of life (15).

In recent years, a number of retrospective studies have investigated the associated risk factors for the development of CPSP after VATS, including body mass index (BMI), history of pain, and acute postoperative pain (13, 14, 16–19). Further, studies have indicated that microRNAs (miRNAs) in the peripheral blood of patients with chronic neuropathic pain are closely associated with chronic neuropathic pain (20–22). Among these, BMI is an indicator used by the National Institute of Health to define a person as underweight, normal weight, overweight, or obese (23). Moreover, studies have confirmed that increased BMI is an independent risk factor for CPSP (24). History of chronic pain refers to the baseline level of pain preoperatively, and retrospective studies have demonstrated that preoperative pain increases the risk of the development of CPSP (14). In addition to preoperative pain, postoperative VAS pain scores on days 1–3 were also positively associated with the occurrence of CPSP after VATS (17). There have been an increasing number of studies implicating miRNAs in their involvement in the occurrence and maintenance of pain, such as the small molecule miR 550a-3p, although its involvement in CPSP has not been confirmed (25).

Notably, patients with CPSP have significantly decreased physical function and poor quality of life after lobectomy (11). Therefore, early prevention and treatment are essential for patients with CPSP to achieve a high quality of life after thoracic surgery (26). Despite several studies in recent years that have investigated risk factors for the development of CPSP after VATS (27–32), they have not been shown to play a predictive role for the occurrence of CPSP. It would be more helpful to improve patient outcomes through the accurate prediction of whether patients who undergo vats will develop CPSP. Nonetheless, to our knowledge, no model has been developed to predict chronic pain after thoracoscopic surgery.

The nomogram prediction model was based on multivariate regression analysis to integrate multiple predictors, thus circumventing the effect of collinearity on the outcome and allowing more accurate prediction of each variable (33). Therefore, to the best of our knowledge, this is the first study to utilize nomogram to establish a prediction model for the occurrence of CPSP after VATS. In this research, we established a nomogram CPSP prediction model for patients with lung adenocarcinoma based on peripheral blood leukocyte miRNAs and clinical characteristics. In the way, we look forward to being able to derive a prediction model with a suboptimal goodness of fit for use by clinicians and, in turn, to optimize clinical decision-making to improve the postoperative quality of life of patients undergoing VATS.

## Methods

### Patients

This prospective study [2019 Fuyi No.2 Ethical Review (No.208)] passed the ethics review of the 90 Second Affiliated Hospital of Fujian Medical University. A prospective design was used to screen 312 patients who underwent VATS between June 2019 and April 2021 (registration ChiCTR2200057092). Based on the intraoperative pathological results, 158 patients with adenocarcinoma were reserved for continued observation. Twelve patients were re-hospitalized within 3 months of discharge, and nine subjects were lost to follow-up for various reasons. After considering the exclusion criteria, 137 patients were included in the final study and divided into two groups according to the presence or absence of CPSP, namely the CPSP group (*n* = 52) and the non-CPSP group (*n* = 85).

Inclusion criteria were as follows: (1) American Society of Anesthesiologists (ASA) grade I–III; (2) No other malignant tumors; (3) No neurological dysfunction; (4) No history of surgery within the past year; (5) Willing to cooperate with follow-up testing and sign the informed consent form.

Exclusion criteria: (1) Transition to open surgery due to various reasons; (2) Postoperative pulmonary complications, secondary surgery, and re-hospitalization within 3 months for other reasons; (3) Failed to complete the study due to various reasons.

### Data collection

All patients were visited in the ward 1 day before surgery to record their general information, including sex, age, body mass index (BMI), ASA grade, sleep quality, anxiety state, history of chronic pain, and presence or absence of hypertension and diabetes mellitus. Data on operation time, duration of drainage, and white blood cell count 1 day before and after surgery and postoperative pain scores on postoperative days 1–3 were recorded. Quantitative information on peripheral leukocyte miRNAs was also collected 1 day before surgery. The occurrence of CPSP (VAS > 0) was recorded *via* telephonic follow-up 3 months after surgery.

### Definitions and questionnaire

The International Association for the Study of Pain (34) and recent studies (1, 3) have defined CPSP as persistent pain for at least 3 months after surgery. That is, the patient had no pain (VSA = 0) at the surgical site before surgery, and VAS relating to pain of the surgical incision was more than 0 three months after surgery. The nature of this pain differs from that of preoperative pain. Patients with chronic pain owing to other causes, such as malignant tumor recurrence or chronic infection, were excluded.

The State–Trait Anxiety Inventory was used to assess anxiety. The Pittsburgh Sleep Quality Index was used to assess sleep quality. The pain score was assessed using the VAS, with scores ranging from 0 (no pain) to 10 (intolerable pain).

### Detection of miRNAs in periph eral leukocytes

Isolation of peripheral leukocytes: the blood of the patient was collected 1 day before the operation, and the fresh whole blood was added into the centrifuge tube containing the separation solution. After centrifugation at 3,500 rpm at 18°C for 20 min, the lower solution was collected. The collected solution was further subjected to red blood cell lysis, the supernatant was discarded, and the leukocyte pellet was collected. Trizol reagent (Invitrogen, Carlsbad, CA, USA) was added, mixed well, and stored at −80°C.

RNA extraction: the leukocytes were lysed, and total RNA was extracted by chloroform extraction, isopropanol precipitation, and ethanol washing and precipitation.

Small RNA library construction and sequencing: three samples were randomly selected from each of the two groups and handed over to Shanghai Ouyi Biomedical Technology Co. Ltd. (Shanghai, China) to complete the construction of the small RNA library and high-throughput sequencing. The difference of miRNA expression between the two groups was detected.

RT-PCR validation: the three miRNAs (miR 550a-3p, miR3613-3p, and miR146a-3p) with the most significant differences were verified by RT-PCR. The method consists of three steps: miRNA isolation (miRNA Kit, TaKaRa, Japan), reverse transcription into cDNA (PrimeScriptTMRT reagent Kit, TaKaRa, Japan), and RT-PCR performed on cDNA (TB Green® Premix Ex TaqTM, TaKaRa, Japan). Three replicate wells were created for each sample. Relative amounts of expression were analyzed using the 2^−ΔΔCt^ algorithm.

### Statistical analysis

The median and value represent discontinuous data and continuous data, respectively. Between-group comparisons between patients with and without CPSP were employed to unpaired *t*-test, Wilcoxon rank-sum test, Pearson's chi-square test, or Fisher's exact test, as appropriate. The least absolute shrinkage and selection operator (LASSO) regression method is used to select data dimensions and prognosis factors. A nomogram model was constructed based on the multivariate unconditional logistic regression analysis results (35). The degree of calibration of the model was assessed using the Hosmer–Lemeshow test, and clinical decision curve analysis (DCA) was performed to analyze the clinical application value of the models (36). Statistical significance was set at *P* < 0.05, and all statistical analyses were performed using R software (version 3.6.2; R Foundation for Statistical Computing, Vienna, Austria).

## Results

**Figure 1:**
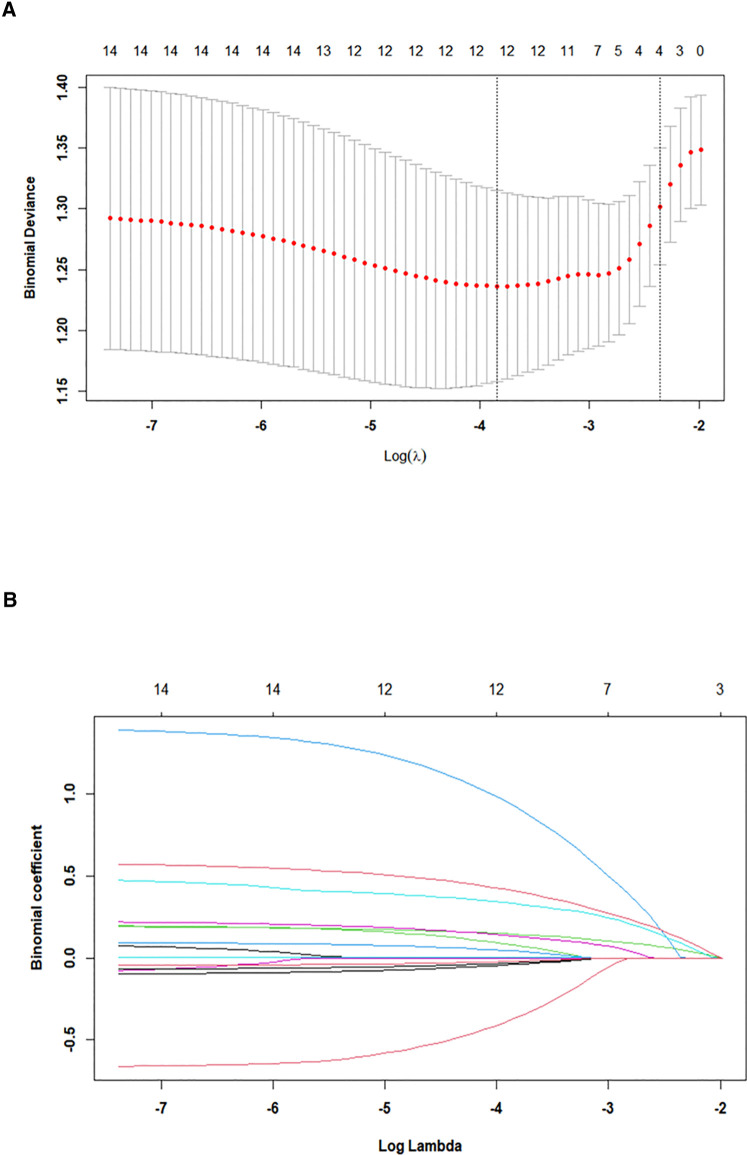
Predictor selection using LASSO regression analysis with 10-fold cross-validation. (**A**) Tuning parameter (lambda) selection of deviance in the LASSO regression based on the minimum criteria (left dotted line) and the 1-SE criteria (right dotted line). (**B**) A coefficient profile plot was created against the log (lambda) sequence. In the present study, predictors were selected according to the 1-SE criteria (right dotted line), where four non-zero coefficients were selected. LASSO, least absolute shrinkage and selection operator; SE, standard error.

A total of 37.9% (52/137) patients included in the study developed CPSP after VATS. Most patients with CPSP presented mild pain, of which 34.6% (18/52) presented with stabbing pain, while the rest presented with dull pain. The basic information and clinical manifestations of all participants included in the study are shown in Table 1. Make use of least absolute shrinkage and selection operator (LASSO) regression technology analyzed that non-zero coefficients of four variables were selected from the 14 variables obtained from patients (Figure 1). These selected variables consist of BMI, history of chronic pain, miR 550a-3p, and VAS score on postoperative day 2 (VAS2d).

**Table 1:** Demographic and clinical characteristics of study participants.

Characteristic	Lung adenocarcinoma patient		*P*-value
	CPSP	non-CPSP	
Sex, *n* (%)			0.588
Male	22 (42.30)	45 (52.94)	
Female	30 (57.69)	40 (47.06)	
Age, *n* (%)			0.098
<60 years	35 (67.31)	45 (52.94)	
≥60 years	17 (32.69)	40 (47.06)	
Body mass index	24.41 ± 3.29	22.69 ± 2.55	<0.001
History of chronic pain			0.021
No	37 (71.15)	74 (87.06)	
Yes	15 (28.85)	11 (12.94)	
Surgery duration (minutes)	207.21 ± 78.59	187.76 ± 74.24	0.148
Drainage time (days)	3.77 ± 1.57	3.40 ± 0.73	0.115
Hospitalization time (days)	4.87 ± 1.58	4.42 ± 0.75	0.065
miRNA146a	1.57 ± 2.89	2.35 ± 4.01	0.225
miRNA550a	1.90 ± 1.47	1.25 ± 0.81	0.001
miRNA3613	1.38 ± 0.76	1.19 ± 0.79	0.174
VAS1d	3.54 ± 1.38	2.94 ± 1.148	0.007
VAS2d	2.96 ± 1.20	2.35 ± 0.10	0.002
VAS3d	2.15 ± 1.04	1.78 ± 0.76	0.016
Pittsburgh Sleep Quality Index	5.38 ± 4.15	6.28 ± 3.63	0.186
State–Trait Anxiety Inventory	29.48 ± 6.66	30.55 ± 7.14	0.383

CPSP, chronic postsurgery pain; VAS, visual analog scale.

**Figure 2:**
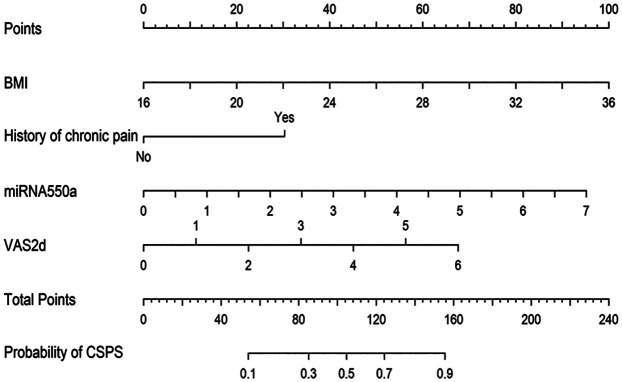
Nomogram for predicting the CPSP risk and its algorithm. First, a point was found for each variable of a lung adenocarcinoma patient after surgery under VATS on the uppermost rule. Then, all scores were added together, and the total number of points was collected. Finally, the corresponding predicted probability of CPSP was found on the lowest rule.

To establish a predictive model for the occurrence of CPSP after VATS, the LASSO regression technique was used to perform multivariate logistic regression analysis of the above four variables. After calculation, the area under the curve (AUC) of nomagram predicted patterns was 0.767 (95% CI: 0.679–0.856). We constructed a nomogram personalized implement for predicting the probability of CPSP after VATS (Figure 2).

**Figure 3:**
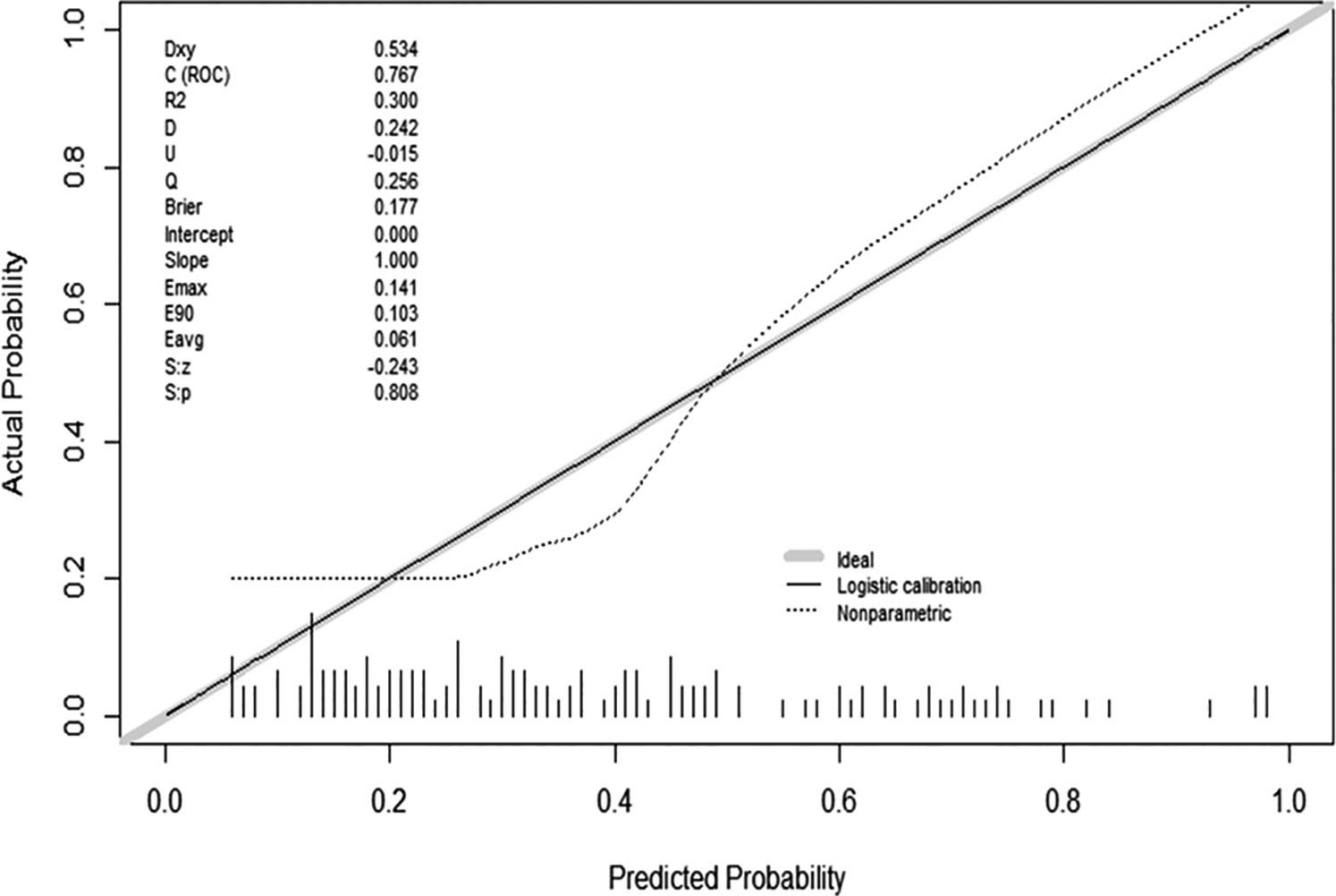
Calibration curve of the predictive model showing the degree of consistency between the predicted probability and observed probability (Hosmer–Lemeshow test, *P* > 0.05, suggesting a high goodness of fit).

The prediction pattern has good calibration effect (Figure 3). The *P*-value from the Hosmer–Lemeshow test was insignificant at 0.808, indicating the predicted value is completely consistent with the observed value without statistical bias.

**Figure 4:**
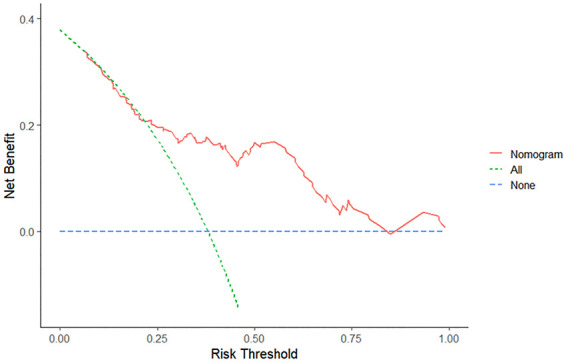
Decision curve analysis of the nomogram. The red solid line represents the nomogram. The decision curve indicates that when the threshold probability of CPSP is between 25% and 100%, the application of this nomogram will add a net benefit compared to the treat-all or treat-none approach.

DCA was performed to evaluate clinical effectiveness of the nomogram. Decision curves revealed that the threshold probability of CPSP after VATS in patients with lung adenocarcinoma was 25%–100% according to the nomogram (Figure 4). Therefore, using the nomogram to predict the risk of CPSP after VATS would be significantly more advantageous than the treat-all or treat-none approach.

## Discussion

In the present study, we established a nomogram to predict the occurrence of CPSP after VATS in patients with lung adenocarcinoma. The nomogram contained four variables—BMI, history of chronic pain, miR 550a-3p, and VAS2d. This predictive model had good discriminative ability, calibration, and clinical usefulness.

The etiology of CPSP after VATS is multifactorial and may involve various relevant factors, including both patient and treatment factors. Some reported predictive and risk factors for the development of CPSP after VATS include BMI, type of VATS, operative time, drainage time, and acute postoperative pain (14, 37, 38). However, none of the above studies proved that the related risk factors could predict CPSP after VATS. Furthermore, in addition to identifying the above similar clinical features as predictors of CPSP development, our prediction model also identified potential predictors at the molecular level.

Studies have revealed that BMI is not associated with postoperative pain after general, orthopedic, or neurospinal surgery (39). However, for VATS, an increase in BMI increases the risk of acute postoperative pain (27). Furthermore, in our prediction model, BMI was identified as a predictor of the occurrence of CPSP after VATS. BMI is an essential indicator of obesity grade. Studies have suggested that the increasing trend of being overweight and obese is associated with increased pain, particularly severe or localized pain, at the population level (28). Increased mechanical loading, body structure changes, and accumulation of biochemical mediators have revealed potential mechanisms linking obesity to pain (29).

A preoperative history of chronic pain, and acute postoperative pain also increases the risk of developing CPSP after VATS. However, this preoperative history of chronic pain may not be related to lung tumors because the patients we included were all early-stage lung adenocarcinomas with no obvious clinical symptoms, and most of the sites involved in their chronic pain history were the lower back, did not involve pain in the chest. Similarly, several clinical trials have reported the correlation between preoperative pain history and CPSP (1, 30). In addition, preoperative pain and acute postoperative pain are independent risk factors for CPSP (14). The increased risk of CPSP after VATS may be due to a preoperative history of pain and acute postoperative pain, leading to sensitization of the central and peripheral sensory systems such that the patient's pain threshold falls (31). Additionally, sensitivity and tolerance to pain vary significantly among individuals; and patients with a history of perioperative pain may be less tolerant to pain (32). Therefore, we should pay attention to postoperative analgesia in high-risk patients who receive VATS to avoid developing acute postoperative pain into CPSP to improve their prognosis.

Finally, we found that elevated miR 550a-3p expression in peripheral leukocytes was a supplementary predictor of the risk of CPSP development after VATS. miRNA are non-protein-coding RNA with critical regulatory roles in various processes under normal and diseased conditions (40). Some studies have demonstrated that miRNA expression discrepancies are closely associated with chronic pain (22, 41). Furthermore, miR 550a-3p has been suggested to promote the proliferation and metabolism of non-small-cell lung cancer cells (42). However, its relevance in pain has not been elucidated, and it may conceivably be related to the potential role of miRNAs in modulating inflammation, pain signal transmission, and protein kinase activity (14). An increasing number of studies have revealed that miRNAs can be utilized as novel biomarkers for the diagnosis, prognosis, and efficacy prediction of various diseases (43, 44). In addition, the detection technology of miRNAs is convenient and fast (44). Based on our nomogram prediction model, we ascertained that miR 550a-3p could predict CPSP occurrence after VATS. Thus, we recommend detecting the expression of miR 550a-3p in VATS patients with high-risk factors, which is more facilitative to predicting CPSP happenings.

However, the limitation is ubiquitous, and this research is no exception. First, this nomogram of the risk of CPSP after VATS was established based on data from a prospectively designed study in the region; therefore, the scope of inclusion is limited by regionality. Second, confounding factors for pain may not have been included in our study. Some of the other complexities of each case, including different surgeons/anesthesiologists, unique incisions, and various effects of different postoperative procedures on pain, were not considered. Finally, we cannot rule out patient-specific health conditions during telephone interviews and surveys, which may have complicated the presentation of pain. Moreover, the potential regulatory mechanism of peripheral blood leukocyte miR 550a-3p expression in CPSP should be further explored. Despite these limitations, this study is the first to develop a nomogram diagnostic method to predict the risk of CPSP after VATS in patients with lung adenocarcinoma.

## Conclusion

The nomogram composed of BMI, history of chronic pain, miR 550a-3p, and VAS2d predicted the risk of CPSP after VATS in patients with lung adenocarcinoma. Further studies are needed to validate our nomogram in different populations. This nomogram may be a valuable complement to the current prediction of CPSP after VATS, which can be advantageous for individual CPSP prediction and clinical decision making after surgery.

## Data Availability

The original contributions presented in the study are included in the article/Supplementary Material, further inquiries can be directed to the corresponding author/s.
